# Construction and Validation of an Autophagy-Related Prognostic Risk Signature for Survival Predicting in Clear Cell Renal Cell Carcinoma Patients

**DOI:** 10.3389/fonc.2020.00707

**Published:** 2020-05-05

**Authors:** Huiying Yang, Mengjiao Han, Hua Li

**Affiliations:** Department of Nephrology, Sir Run Run Shaw Hospital, Zhejiang University School of Medicine, Hangzhou, China

**Keywords:** clear cell renal cell carcinoma, TCGA, prognostic risk signature, autophagy-related genes, overall survival, prognostic outcome, least absolute shrinkage and selection operator (LASSO) Cox regression, effect prediction of precise treatments

## Abstract

**Background:** Clear cell renal cell carcinoma (ccRCC) is a common type of malignant tumors in urinary system. Evaluating the prognostic outcome at the time of initial diagnosis is essential for patients. Autophagy is known to play a significant role in tumors. Here, we attempted to construct an autophagy-related prognostic risk signature based on the expression profile of autophagy-related genes (ARGs) for predicting the long-term outcome and effect of precise treatments for ccRCC patients.

**Methods:** We obtained the expression profile of ccRCC from the cancer genome atlas (TCGA) database and extract the portion of ARGs. We conducted differentially expressed analysis on ARGs and then performed enrichment analyses to confirm the anomalous autophagy-related biological functions. Then, we performed univariate Cox regression to screen out overall survival (OS)-related ARGs. With these genes, we established an autophagy-related risk signature by least absolute shrinkage and selection operator (LASSO) Cox regression. We validated the reliability of the risk signature with receiver operating characteristic (ROC) analysis, survival analysis, clinic correlation analysis, and Cox regression. Then we analyzed the function of each gene in the signature by single-gene gene set enrichment analysis (GSEA). Finally, we analyzed the correlation between our risk score and expression level of several targets of immunotherapy and targeted therapy.

**Results:** We established a seven-gene prognostic risk signature, according to which we could divide patients into high or low risk groups and predict their outcomes. ROC analysis and survival analysis validated the reliability of the signature. Clinic correlation analysis found that the risk group is significantly correlated with severity of ccRCC. Multivariate Cox regression revealed that the risk score could act as an independent predictor for the prognosis of ccRCC patients. Correlation analysis between risk score and targets of precise treatments showed that our risk signature could predict the effects of precise treatment powerfully.

**Conclusion:** Our study provided a brand new autophagy-related seven-gene prognostic risk signature, which could perform as a prognostic indicator for ccRCC. Meanwhile, our study provides a novel sight to understand the role of autophagy and suggest therapeutic strategies in the category of precise treatment in ccRCC.

## Introduction

Renal cell carcinoma (RCC) is one of the most common types of malignant tumors, which accounts for ~2% of all kind of cancer diagnoses and deaths worldwide. The average annual incidence cases of RCC are about 295,000 worldwide (1). Clear cell renal cell carcinoma (ccRCC) is the main subtype of RCC and occupies about 80–90% of all (2, 3).

In clinical work, it is a vital and hard task to estimate the long-term outcome of tumor patients and make therapeutic decisions accordingly. TNM staging is a classical method for assessing the prognostic outcome of tumor patients and has been employing for almost 100 years. But, it seems that TNM staging could be limited or incomplete and patients in the same condition of TNM stage may come to entirely distinct outcomes. What's more, although most patients were diagnosed with localized disease, ~30% of early-stage patients still experience local recurrence or distant metastasis after surgery (4). Thus, more effective and precise methods are needed for outcome predicting.

With the rapid development of high-throughput next-generation sequencing, gene microarray technique, machine leaning, and bioinformatics analyses, researchers have found risk signature, which consists of clinical features and molecular characteristics, could act as a new approach for estimating the prognosis outcome of ccRCC patients. Several risk signatures have shown satisfactory effects in outcome predicting, such as immune-associated gene signature (5, 6), long non-coding RNA based gene signature (7), nomograms (8), and so on. Researchers hope that these novel approaches would help doctors make more appropriate estimations, take more personalized therapeutic strategies, increase the curative ratio for malignant tumors, and extend the overall survival (OS) as a result for tumor patients.

Autophagy is a lysosomal degradation pathway in cellular process, which is supposed to protect cells and tissues from stressors in normal physiological processes. The proper processes of autophagy are indispensable for survival, differentiation, development, and homeostasis. Recent researches have revealed that autophagy also plays an essential role in various pathological processes, especially in the pathophysiology of malignant tumor. Previous studies have demonstrated that inappropriate processes of autophagy would support the growth of tumor and some antineoplastic clinical trials have employed autophagy inhibiting as a novel therapeutic approach (9).

However, although quite a lot studies have investigated the patterns of gene expression of ccRCC from different aspects and have constructed several prognostic risk signatures previously, there is no such research designed to clarify the expression pattern of autophagy-related genes (ARGs) or attempted to develop a prognostic risk signature with ARGs in ccRCC.

Our study aims at constructing a risk signature for ccRCC with the expression profile of ARGs. We obtained the whole gene expression profiles of ccRCC and normal control from the cancer genome atlas (TCGA) database and extracted the expression profile of all ARGs. Then, we identified differentially expressed genes (DEGs) between ccRCC and normal control from all ARGs and performed enrichment analyses on DEGs to clarify the autophagy-related aberrant biological functions in ccRCC. On the basis of a training dataset, we analyzed the correlation between the expression levels of ARGs and overall survival (OS) of patients, performed least absolute shrinkage, and selection operator (LASSO) Cox regression with some ARGs of significant correlation with OS, and constructed an ARG-based risk signature as a result. We then validated the reliability of our risk signature with survival analysis, receiver operating characteristic (ROC), univariate cox regression, and correlation analysis of risk group, and clinical traits on a test dataset. We conducted single-gene gene set enrichment analysis (GSEA) on genes of our risk signature, respectively, to explore the roles of these genes in ccRCC. Finally, we investigated the correlation between our risk signature and the expression level of targets of immunotherapy and targeted therapy in order to help physicians predict the effects of precise treatments and decide curative strategies more efficiently.

With this novel risk signature, we expect to give more helpful guidance for clinical work and a new point of mechanism research for ccRCC.

## Materials and Methods

### Data Collection

**Figure 1:**
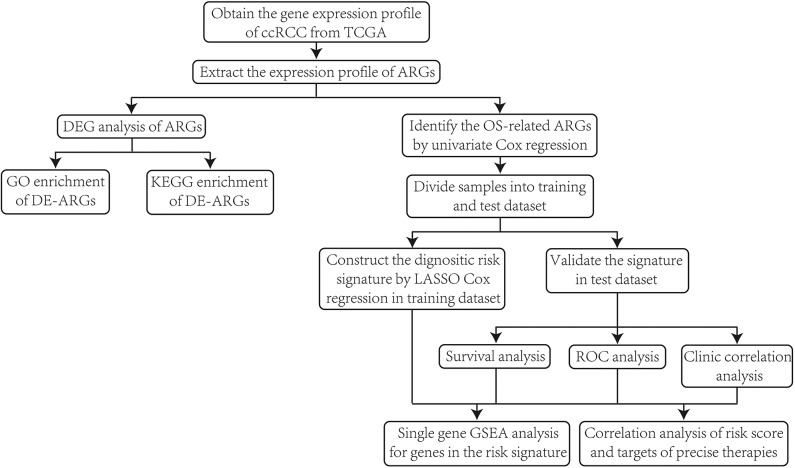
The flow chart of the overall procedures.

First of all, we illustrated the overall procedures of our study in a flow chart (Figure 1).

We collected all transcriptome profiles of ccRCC available in the database of TCGA (https://portal.gdc.cancer.gov/) at 21th September, 2019. Our study included the expression profile of 72 normal samples and 539 ccRCC samples. We collected the corresponding clinical information of these patients from TCGA at the same time. The clinical information included gender, age, tumor grade, pathological stage, TNM stage, follow-up time, survival status. Details of clinical information are given in Table S1, and samples missing any clinical characteristics were excluded in the following analyses. As the lymphatic metastasis was not regarded as an important evaluation criterion for ccRCC clinically, and the status of lymphatic metastasis is missing in most samples, we didn't take the status of lymphatic metastasis into consideration in the current study. For drawing more convincing conclusions, we excluded samples with follow-up time shorter than 3 months.

### Collection of Autophagy-Related Genes

The Human Autophagy Database (HADb, http://www.autophagy.lu/) provides a complete and real-time updated list of human genes related with the biological processes of autophagy reported in PubMed or other common databases (10). We got 231 ARGs in all form HADb, and the expression levels of 209 genes were available in the expression profile from TCGA (The gene list are given in Table S2).

### Differentially Expressed Analysis of Autophagy-Related Genes

To confirm if the expression levels of ARGs have changed in tumor tissue of ccRCC compared with normal control, we conducted differentially expressed analysis on all ARGs based on wilcoxon test with limma package (11) under R environment (version 3.6.1). The cut-off criterion for differentially expressed genes (DEGs) was set as *p* < 0.01 and *|log*_*FC*_*|* > 0.5. The results are displayed with pheatmap package (https://cran.r-project.org/web/packages/pheatmap/index.html) under R environment. Here, we renamed the DEGs extracted from ARGs as differentially expressed autophagy-related genes (DE-ARGs).

### Enrichment Analyses of Differentially Expressed Autophagy-Related Genes

We performed Gene Ontology (GO) (12) enrichment analysis and Kyoto Encyclopedia of Genes and Genomes (KEGG) (13) pathway enrichment analysis to explore the main roles of disordered DE-ARGs in ccRCC. The cut-off criterion was *p* < 0.01 and Benjamin-Hochberg adjusted *p* < 0.01 for both of GO and KEGG enrichment analyses. The analyses and the visualization of results were conducted under R environment with clusterProfiler package (14).

### Construction of ARG-Based Prognostic Risk Gene Signature

Firstly, we conducted a time-dependent univariate Cox regression with survival package under R environment for screening out genes of significant prognostic predicting value for ccRCC from all ARGs. Genes of significant correlations with OS (*p* < 0.01) were considered as prognosis-related genes. Meanwhile, the prognosis predictors were distinguished between risky genes and protective genes with HR value (HR >1 means risky genes and HR <1 means protective genes).

Then, for constructing and validating the gene signature, we divided the total of 539 tumor samples into training dataset and test dataset randomly according to a proportion of 7:3 (377 samples in training dataset and 163 samples in test dataset, information of the groups are available in Table S3).

Finally, we adopted least absolute shrinkage and selection operator (LASSO) Cox regression on the training dataset with top 10 OS-related ARGs identified by univariate Cox regression (we selected only 10 genes for LASSO Cox regression to avoid potential overffiting of the signature). LASSO Cox regression was employed for two purposes. The first one is to establish a prognostic predictive module (risk signature), which could give a regression coefficient to each gene and then calculate a risk score for each patient. The other one is to filter genes of high correlations with each other to prevent overfitting of the module. The final formula of the risk signature was given as risk score = expression level of Gene1^*^β1 + expression level of Gene2^*^β2 +…+expression level of Genen^*^ βn, in which β represents the regression coefficient of each variable.

We also performed survival analysis on selected the genes to explore the effects of single gene on the OS of ccRCC patients.

### Verification of the Risk Signature

To verify the validity and robustness of the LASSO Cox regression risk signature, we calculated risk score for each patient in the test dataset and separated them into low-risk and high-risk groups based on the median of risk score. Then we conducted overall survival analysis (conducted by Kaplan–Meier method with a two-sided log-rank test, with survival package under R environment) between the two groups to explore the difference of OS between the two risk groups. Further, we assessed the efficiency of OS-predicting of our risk signature by operating receiver operating characteristic (ROC) curve (with survivalROC package under R environment). At the same time, we analyzed the correlation between risk score and clinical traits to confirm the validity of our risk signature.

We then performed univariate and multivariate Cox regression analyses to verify the prognostic value of the risk score. We took age, gender, tumor grade, pathological stage, TNM stage (lymphatic metastasis excluded) as candidate risk factors for regression analyses. We evaluated if all these factors are risk factors for worse outcomes by univariate Cox regression analysis, and further determined if the risk score calculated by our risk signature could be utilized for predicting the prognosis of ccRCC patients independently.

### Function Analyses of Genes in the Risk Signature

In order to clarify the potential roles of genes included in our risk signature in ccRCC, we performed single-gene gene set enrichment analysis (GSEA) (15) on the genes, respectively. We first divided all samples into high-expression group and low-expression group by the expression level of a single gene, and then applied GSEA to identify the different signal pathways between the two groups. We performed GSEA on each gene one by one with a desktop application for GSEA (16). The cut-off criterion for statistically significant terms was decided as *p* < 0.05.

### Exploration of the Association Between Risk Score With Targets of Immunotherapy and Targeted Therapy

Immunotherapy and targeted therapy are revolutionary and effective approaches for ccRCC treatment. Food and Drug Administration (FDA) have approved kinds of precise therapies in the past decades (17). Here, we analyzed the correlation of our risk score with the therapy-related targets by Pearson's correlation analysis and attempted to predict the treatment effect with our risk score. The therapy targets are listed as follow: programmed cell death 1 (*PD-1*, also known as *PCDC1*), programmed cell death ligand 1 (*PD-L1*, also known as *CD274*), vascular Endothelial Growth Factor Receptor (*VEGFR1*, also known as *FLT1*), vascular Endothelial Growth Factor Receptor 3 (*VEGFR3*, also known as *FLT4*), mammalian target of rapamycin (*mTOR*), platelet-derived growth factor receptor alpha (*PDGFRA*), platelet-derived growth factor receptor beta (*PDGFRB*), *KIT* proto-oncogene (*KIT*), Fms-like tyrosine kinase 3 (*FLT3*), ret proto-oncogene (*RET*), and *MET* proto-oncogene (*MET*).

## Results

### Differentially Expressed Analysis and of Autophagy-Related Genes

**Figure 2:**
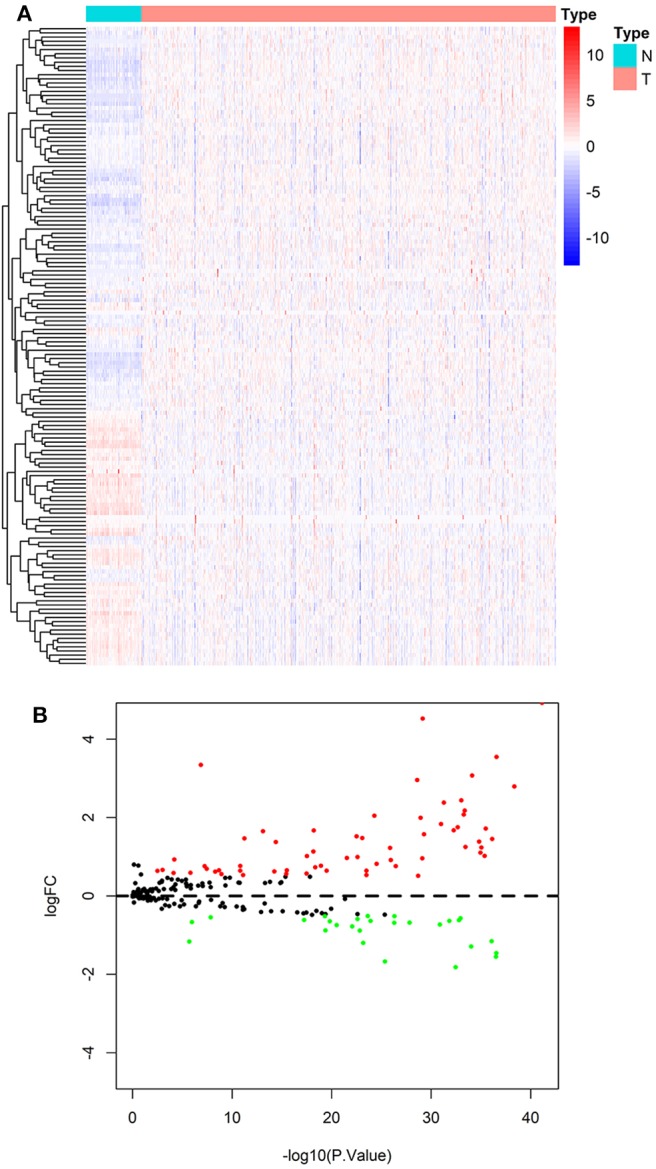
Result of differentially expressed analysis on ARGs. **(A)** A heatmap of DE-ARGs. Each line represents a DE-ARG and each row means a sample. The expression levels of genes are displayed with colors in each cell (red for high and blue for low). **(B)** A volcano plot of the logFC and statistical significance of all ARGs. Red plots represent up-regulated DE-ARGs and green ones represent down-regulated ones. Black plots are genes didn't reach the criteria of DEGs.

According to the screening criteria of DEGs, 89 of the 231 ARGs showed significant alterations of expression levels in ccRCC compared with normal control, including 61 up-regulated and 27 down-regulated genes, respectively. The results are shown in a heatmap (Figure 2A) and a volcano plot (Figure 2B). Details such as log_2_-fold change (logFC) and statistical significance are given in Table S4.

### GO and KEGG Pathway Enrichment Analyses on DE-ARGs

**Figure 3:**
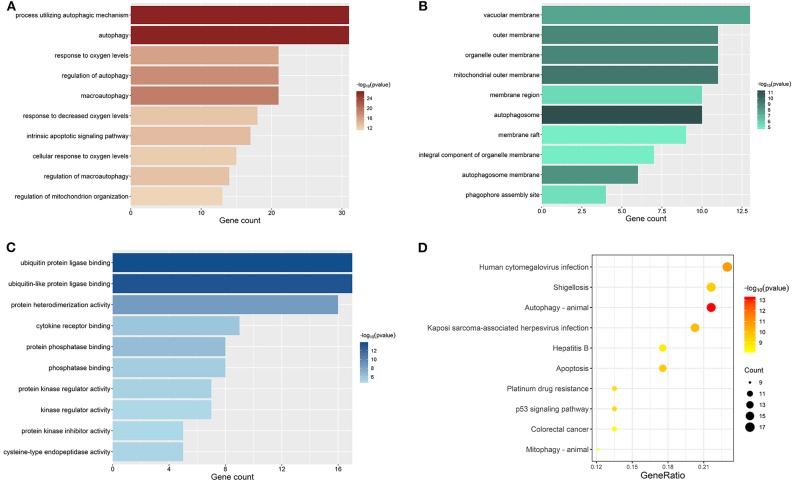
Results of enrichment analyses of DE-ARGs. The color represents the statistical significance of the term. The length indicates the counts of enriched genes. **(A)** Top 10 significant GO-BP terms. **(B)** Top 10 significant GO-MF terms. **(C)** Top 10 significant GO-CC terms. **(D)** Top 10 significant KEGG signal pathways.

All genes enrolled in our research were ARGs, and some of them showed no signs of changes between ccRCC and normal control. We explored the aberrant biological functions by performing enrichment analyses on DE-ARGs. GO enrichment analysis includes three categories: biological process (BP), cellular component (CC), and molecular function (MF). The top 10 significant terms were shown in Figure 3 and all terms are available in Table S5. According to the results of GO-BP, some terms were not specific conceptions, such as “autophagy” (gene count = 31, *p* = 3.61E-27), “process utilizing autophagic mechanism” (gene count = 31, *p* = 3.61E-27), and “macroautophagy” (gene count = 21, *p* = 9.19E-20). But some terms still pointed to specific biological processes playing important roles in the development of tumor, such as “response to oxygen levels” (gene count = 21, *p* = 1.41E-16), “response to oxidative stress” (gene count = 16, *p* = 3.43E-10), “response to starvation” (gene count = 11, *p* = 7.82E-10). The results of KEGG pathway enrichment analysis showed that the dysfunction of the autophagy-related genes may contribute to the drug resistance of ccRCC, such as “platinum drug resistance” (gene count = 10, *p* = 9.44E-10), “endocrine resistance” (gene count = 9, *p* = 2.43E-07). Some other well-known signal pathways in tumor pathophysiology were also enriched, such as “*p53* signaling pathway” (gene count = 10, *p* = 8.21E-10) and “HIF-1 signaling pathway” (gene count = 10, *p* = 4.98E-08).

### Identification of Prognosis-Related ARGs by Univariate Cox Regression

**Figure 4:**
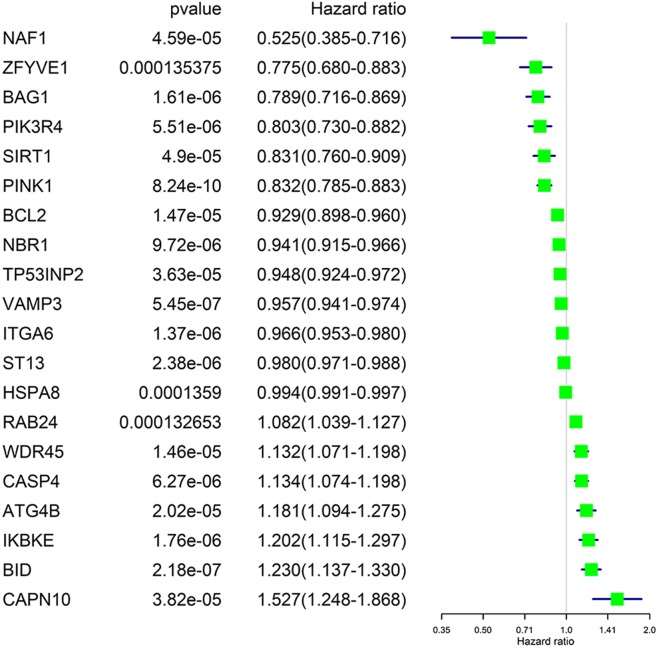
Significance and Hazard ratio values of OS-related ARGs in univariate Cox regression.

We performed a univariate Cox regression analysis on all ARGs (rather than DE-ARGs) to identify genes of significant correlation with OS. The results showed that 38 genes were significantly related with OS of ccRCC patients (*p* < 0.01, details are given in Table S6). The top 20 significant genes were displayed in Figure 4 with their Hazard ratio values. We could come to a conclusion that 13 of the 20 OS-related ARGs were protecting factors and seven were risk factors. What's more, we found that not all OS-related ARGs were DE-ARGs.

### Construction of the ARG-based Prognostic Risk Gene Signature by LASSO Cox Regression

As OS-related ARGs may function through interaction with each other rather than independently, we performed the LASSO Cox regression with the top 10 significant OS-related ARGs to determine the real OS-affecting factors and construct a risk signature on the training dataset. When the partial likelihood deviance reached minimum, the corresponding count of factors was seven and log (Lambda) was about −3.8 (Figure S1A). The coefficients are shown in Figure S1B. The formula for calculating the risk score according to the risk signature was given as follow (more precise coefficients, the formula, and the median of risk score are given in Table S7):

$$\begin{array}{l} \text{Riskscore}=-0.0953^{*}{\text{Exp}}_{PINK1}+0.0486^{*}{\text{Exp}}_{BID}\\ \;-0.0068^{*}{\text{Exp}}_{VAMP3}-0.0492^{*}{\text{Exp}}_{BAG1}-0.0017\\ {\;}^{*}{\text{Exp}}_{ST13}-0.0279^{*}{\text{Exp}}_{PIK3R4}+0.0223^{*}\;{\text{Exp}}_{CASP4} \end{array}$$

**Figure 5:**
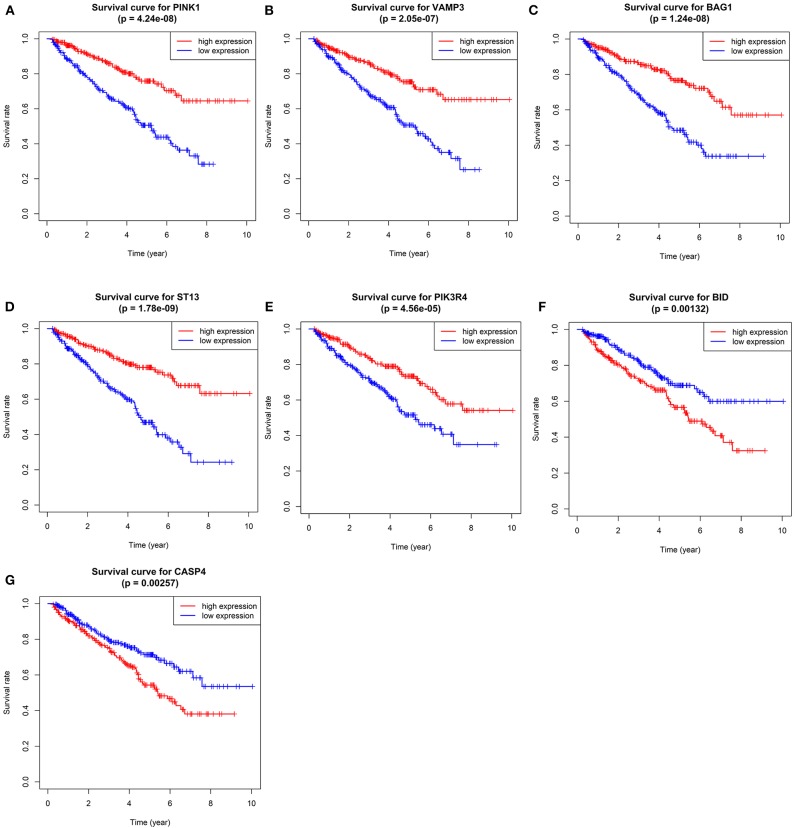
Kaplan–Meier overall survival (OS) curves for ccRCC patients assigned to groups of high and low expression level of based on the seven genes, respectively. (**A–G** shows the results of *PINK1, VAMP3, BAG1, ST13, PIK3R4, BID*, and *CASP4*, respectively).

We performed survival analyses on the seven genes and exhibited the results in Figure 5.

**Figure 6:**
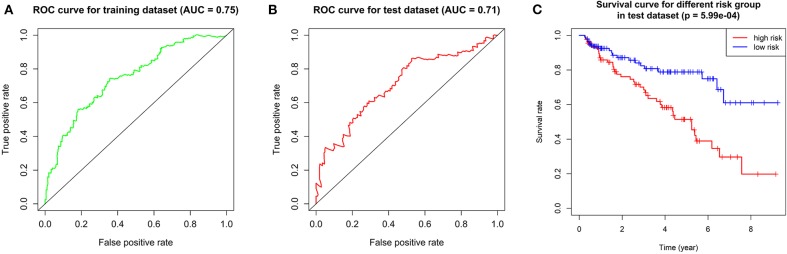
Validation of the prognostic gene signature. **(A)** ROC curve showing the predictive efficiency of the risk signature in training dataset. **(B)** Kaplan–Meier overall survival (OS) curve for patients in test dataset assigned to groups of high risk and low risk based on our signature. **(C)** ROC curve showing the predictive efficiency of the risk signature in test dataset.

We calculated the risk score for each patient in training dataset and divided them into high-risk group and low-risk group based on the median of risk score. Patients in high-risk group are deemed to be at higher risk of death. Then we assessed the prediction efficiency of the risk signature by operating a ROC curve, which revealed that the risk score could predict the 5-year survival rate for ccRCC patient effectively (AUC = 75%, Figure 6A).

### Validation of the Risk Signature

We verified the robustness of our risk signature with the test dataset. We calculated the risk score for patients and divided them into high-risk group and low-risk group in test dataset with the same formula mentioned above.

We performed overall survival analysis on the two groups. Patients in low-risk group had obviously better outcome (*p* = 5.99E-4) and had higher 5-year survival rate (49% in high-risk group and 80% in low risk group, respectively, Figure 6C). We also operated ROC analysis in test dataset to find a stable predictive ability of our risk signature (AUC = 71%, Figure 6B).

As the overall survival analysis and ROC analysis have demonstrated the satisfactory robustness of the prognostic value of our risk signature, we put the training dataset and test dataset together again for subsequent analyses.

**Figure 7:**
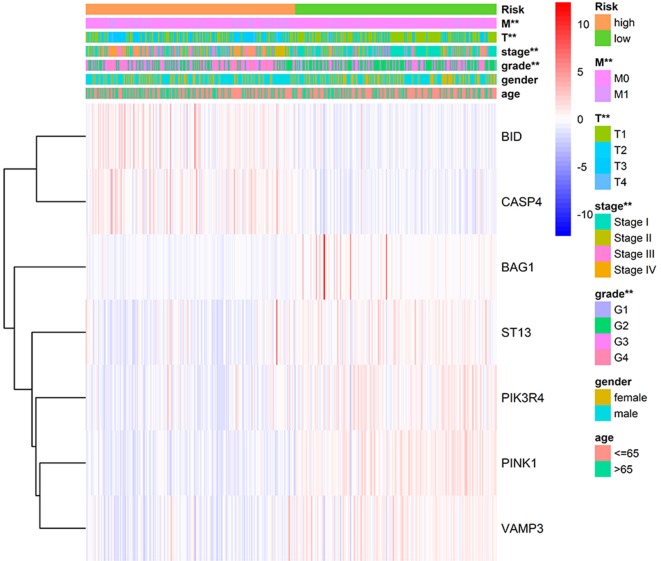
Correlation of risk group and clinical traits.

We applied correlation analysis between risk group and clinicopathologic features with chi-square test to find high risk score is closely related to tumor grade (*p* = 9.302E-9), pathological stage (*p* = 1.675E-11), T stage (*p* = 1.256E-10), distant metastasis (*p* = 2.251E-5), but isn't related to gender and age (Figure 7, Table S8). Moreover, the expression patterns of the seven genes were quite similar in the same group and differed distinctly between different groups, hinting the accuracy of the risk signature.

### Prognostic Value Verification of the Risk Signature by Cox Regression Analyses

**Figure 8:**
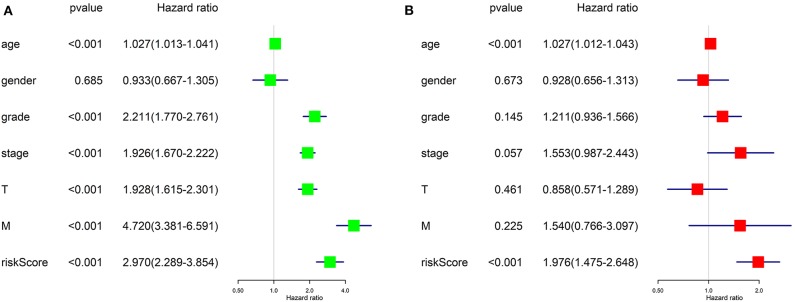
Results of Cox regression for risk factors for ccRCC. **(A)** Result of Univariate Cox regression. **(B)** Result of multivariate Cox regression.

Univariate Cox regression (Figure 8A) revealed that advanced age, higher tumor grade, pathological stage, T stage, distant metastasis, and higher risk score assessed by our risk signature were risk factors for worse prognostic outcome. It goes without saying that the former five factors would affect the prognostic outcome of patients with tumor, while the results proposed that our risk score is also a risk factor for ccRCC (HR = 2.970, *p* < 0.001).

Further, we applied multivariate Cox regression for determining if the risk score could be utilized as an independent prognosis predictor for ccRCC patients. The result (Figure 8B) revealed that only advanced age (*p* < 0.001) and our risk score (*p* < 0.001) remained significantly associated with OS of ccRCC patients. The results of Cox regression analyses confirmed that the risk score derived from ARGs could act as an independent prognosis predictor for ccRCC patients. The efficiency of predicting would not be affect by any clinicopathologic features of the tumor.

### Single-Gene Gene Set Enrichment for the Seven Genes

**Figure 9:**
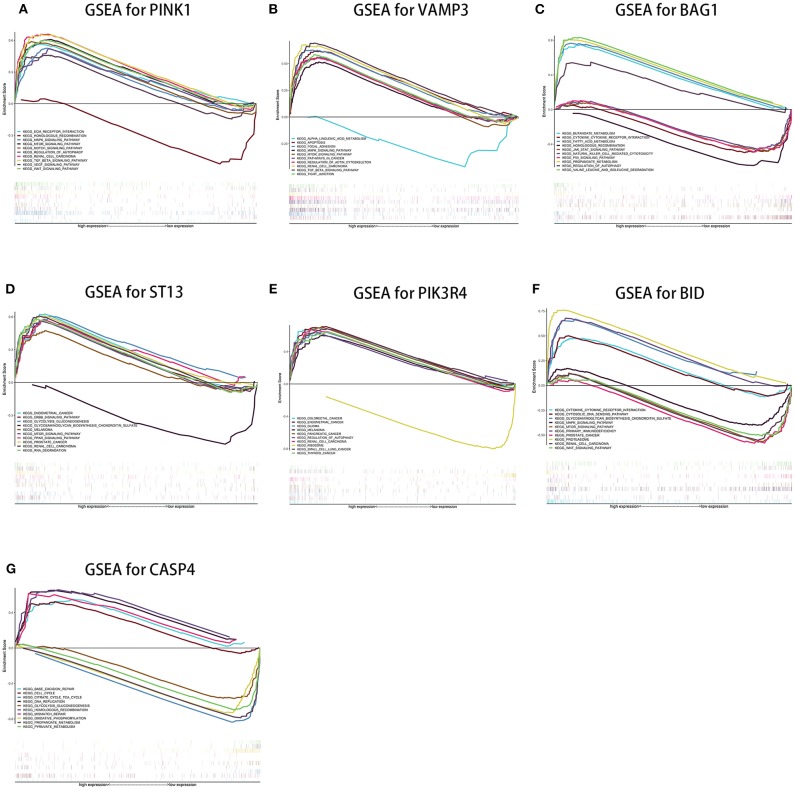
Results of single-gene GSEA of seven genes in our risk signature in ccRCC. (**A–G** shows the results of *PINK1, VAMP3, BAG1, ST13, PIK3R4, BID*, and *CASP4*, respectively).

Single-gene GSEA of the seven genes has shown the potential roles of the genes in ccRCC (Figure 9). We exhibited 10 signal pathways in each figure of Figure 9. If there are more than 5 signal pathways in both of up-regulated ones and down-regulated ones, we exhibit 5, respectively. Otherwise, if the up-regulated signal pathways are <5, we show more down-regulated ones instead. For example, *PINK1* was identified as a protective factor for ccRCC, the signal pathways of “*TGF-*β signal pathway,” “*mTOR* signal pathway,” “*VEGF* signal pathway” are up-regulated in high-expression group (namely, relatively low risk group) of *PINK1*, and “Homologous recombination” down-regulated in high-expression group of *PINK1*. The result was in accordance with the conclusions given in the next portion that low-risk group may response better to therapies targeting *VEGF1, VEGF3, mTOR*.

### Effectiveness Predicting of Immunotherapy and Targeted Therapy With Our Risk Score

**Figure 10:**
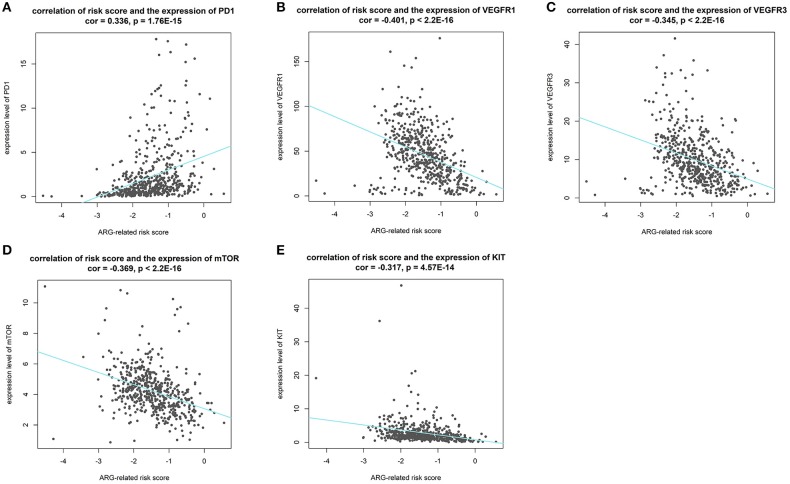
Results of correlation analysis between our risk score and expression level of targets of precise treatment in ccRCC. (**A–E** shows the results of *PD-1, VEGFR1, VEGFR3, mTOR, KIT*, respectively).

Results of Pearson's correlation analysis indicated that our risk score was significantly correlated with the mRNA expression level of *PD-1* (cor = 0.336, *p* = 1.76E-15), *VEGFR1* (cor = −0.401, *p* < 2.2E-16), *VEGFR3* (cor = −0.345, *p* < 2.2E-16), *mTOR* (cor = −0.369, *p* < 2.2E-16), and *KIT* (cor = −0.269, *p* = 3.788e-10). Results are visualized in Figure 10. The results reveal that patients with higher risk score might response better to therapies targeting *PD-1*, and patients with lower risk score might response better to therapies targeting *VEGFR1, VEGFR3, mTOR*, and *KIT*.

## Discussion

In the current study, we used the expression profile of the tumor tissue of ccRCC patients as well as normal control downloaded from TCGA database to construct an autophagy-related prognostic gene risk signature. We obtained 231 ARGs from HADb database and extracted an expression profile contains only ARGs. We performed differentially expressed analysis on ARGs and screened out 89 DE-ARGs. Enrichment analyses on these DE-ARGs elucidated that the irregular biological processes are mainly involved in themes as follow: the response of tumor toward oxygen level and starvation, the resistance of tumor to some treatment strategies, and several well-known tumor-related pathways, such as p53 signaling pathway. We then identified OS-related ARGs with univariate Cox regression analysis. With the top 10 OS-related ARGs, we developed a novel prognostic risk signature trained by LASSO Cox regression. With the risk signature, we calculated the risk score for each patient and divided all patients into high-risk group and low-risk group. The result of overall survival analysis revealed that the OS of high-risk group was significantly poorer than that of low-risk group, and ROC analysis reflected a satisfactory accuracy of the risk signature (AUC = 75% in training dataset and 71% in test dataset). Meanwhile, clinic correlation analysis showed that the risk group was closely related to tumor grade, pathological stage, T stage, and distant metastasis. We validated the reliability of our risk signature by the three approached above. Furthermore, multivariate Cox regression found that the risk score could act as a satisfactory independent predictor for the prognosis of ccRCC patients. In order to clarify the potential roles of the seven genes in ccRCC, we conducted single-gene GSEA for each gene. Moreover, we analyzed the correlation between our ARG-related risk score with targets of immunotherapy and targeted therapy to find our risk score significantly correlated with the mRNA expression level five therapy targets, providing potential guidance for personalized treatments. In all, our work implied that dysfunction of autophagy plays a vital role in the process of ccRCC. Most importantly, we have developed a robust ARG-based prognostic risk signature for ccRCC for the first time, which is of great relevance to clinicians and patients and may provide a more precise estimate method for the prognosis of ccRCC patients and more individualized treatment strategies accordingly.

It is worth mentioning that we conduct LASSO Cox regression with not only DE-ARGs, but also ARGs weren't included in DEGs. Interestingly, we found some genes influence the OS of patients strongly in survival analysis but didn't reach the criterion of DE-AGRs. For example, the statistical significance of *ST13* in survival analysis was *p* = 1.78E-09, and the 5-year survival rate different sharply in high-risk and low-risk groups, but the logFC of the gene was low as −0.118. On the contrary, some genes were DE-ARGs but their influences on OS were not such significant. This phenomenon reminds us that we should not judge the importance of genes by differentially expressed analysis and should take all OS-related genes into consideration while establishing a risk signature.

Autophagy is a relatively conserved process in normal physiological processes (18). The relationship between autophagy and cancer is still controversial. It is reported that the roles of autophagy are dynamic during different stages of the initiation and progression of cancer. In the initial stage of tumorigenesis, autophagy is a suppressor toward tumor initiation, and cancer progression. While, in the advanced stage of tumor, autophagy would act as a protective factor for promoting survival, growth, and aggressiveness (19). Briefly, we have sufficient reasons to believe that regulating the status of autophagy properly would help us to carve a new path in the treatment of ccRCC.

Our risk signature is comprised of seven genes, including *PINK1, BID, VAMP3, BAG1, ST13, PIK3R4*, and *CASP4*. Our results found *PINK1, VAMP3, BAG1, ST13*, and *PIK3R4* as protect factors and *BID, CASP4* as risk factors for ccRCC. Regrettably, no related studies have explained the roles of all seven genes in ccRCC. But there still exist some papers about the function of these genes in other kind of tumors. *PINK1* controls stress-related and metabolism-related functions in various tumors (20, 21). *BID* plays a major role in apoptosis and its dysfunction underlies the process of carcinogenesis (22). *VAMP3* functions during the degradation of extracellular matrix and subsequent cellular invasion in tumor (23). *BAG1* is a multifunctional anti-apoptotic protein and is involved in the regulation of some cytokines, such as epidermal growth factor receptor and hepatocyte growth factor receptor (24). *ST13* is an inhibitor of cell proliferation, colony formation, and cell migration and could induce apoptosis in colorectal cancer (25, 26). The function of *CASP4* is related to the cell migration and cell-matrix adhesion and down-regulation of *CASP4* results in more severe tissue invasion (27). No studies explained the role of *PIK3R4* in tumor yet.

Nonetheless, there are still some limitations in our study. First, we established the prognosis signature only with the expression profile of ccRCC obtained from TCGA. Although we have divided all date into two parts of training dataset and test dataset, and then validated the robustness of the prognostic signature established in training dataset with the data in test dataset, our conclusion would be more convincing with validation in independent external datasets. We would like to complete this portion of work after collecting some samples and data of expression profile by ourselves. Secondly, the biological functions of the genes making up the prognostic signature are needed to be explored deeply in ccRCC.

## Conclusion

Briefly, our work established an autophagy-related prognostic risk signature for the first time and validated its reliability successfully. Our work might provide a novel method for prognosis predicting and personalized therapeutic strategies selecting. Meanwhile, our study may help researchers to explore the molecular mechanisms behind ccRCC from a brand new insight.

## Data Availability Statement

Publicly available datasets were analyzed in this study, these can be found in The Cancer Genome Atlas (https://portal.gdc.cancer.gov/).

## Author Contributions

HY and MH conceived, designed, and conducted the study, as well as wrote the manuscript. All authors reviewed the manuscript and participated in the language modification.

## Conflict of Interest

The authors declare that the research was conducted in the absence of any commercial or financial relationships that could be construed as a potential conflict of interest.

## Supplementary Material

Supplementary Figure 1 Establishment of the risk signature by LASSO Cox Regression. **(A)** Determination of the counts of genes included in the signature. **(B)** Determination of the coefficients of the genes.

Table S1 Clinical information of ccRCC samples included in our research.

Table S2 Gene symbols of ARGs available in TCGA (209 genes in all).

Table S3 Division of training and test datasets.

Table S4 Information of the results of DEG analysis in ARGs.

Table S5 GO and KEGG enrichment analyses.

Table S6 Statistical significance and HR of OS-related ARGs.

Table S7 ARGs and their coefficients in our signature.

Table S8 χ^2^ squared test of clinical traits and risk score.
