# Supplementary figures and images for: Construction and Validation of an Autophagy-Related Prognostic Risk Signature for Survival Predicting in Clear Cell Renal Cell Carcinoma Patients
Source: Front Oncol. 2020 May 5;10:707. doi: 10.3389/fonc.2020.00707 (PMC7214632; doi:10.3389/fonc.2020.00707)

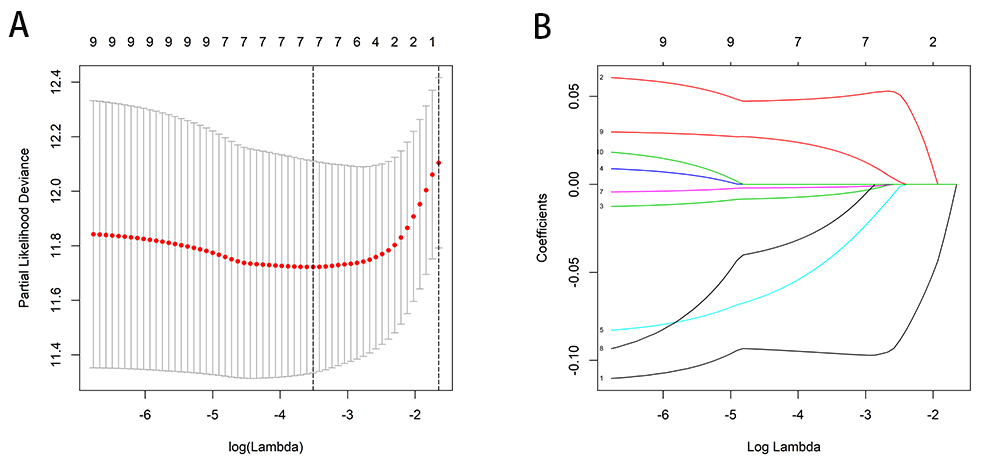

Supplement: Supplementary Figure 1 — Establishment of the risk signature by LASSO Cox Regression. (A) Determination of the counts of genes included in the signature. (B) Determination of the coefficients of the genes. [file Image_1.JPEG]
